# Supplementary figures and images for: Deconstructing the Polymerase Chain Reaction II: an improved workflow and effects on artifact formation and primer degeneracy
Source: PeerJ. 2019 Jun 14;7:e7121. doi: 10.7717/peerj.7121 (PMC6573857; doi:10.7717/peerj.7121)

Supplemental Figure S1A

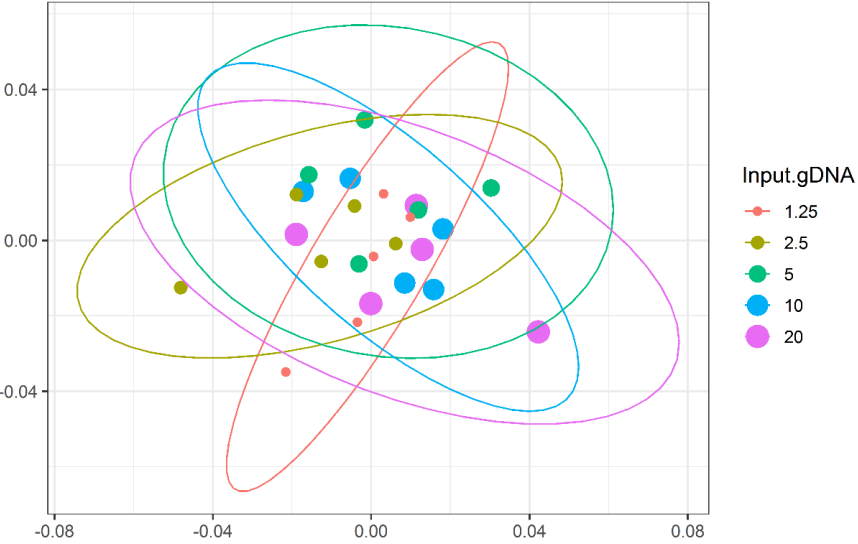

Supplemental Figure S1B

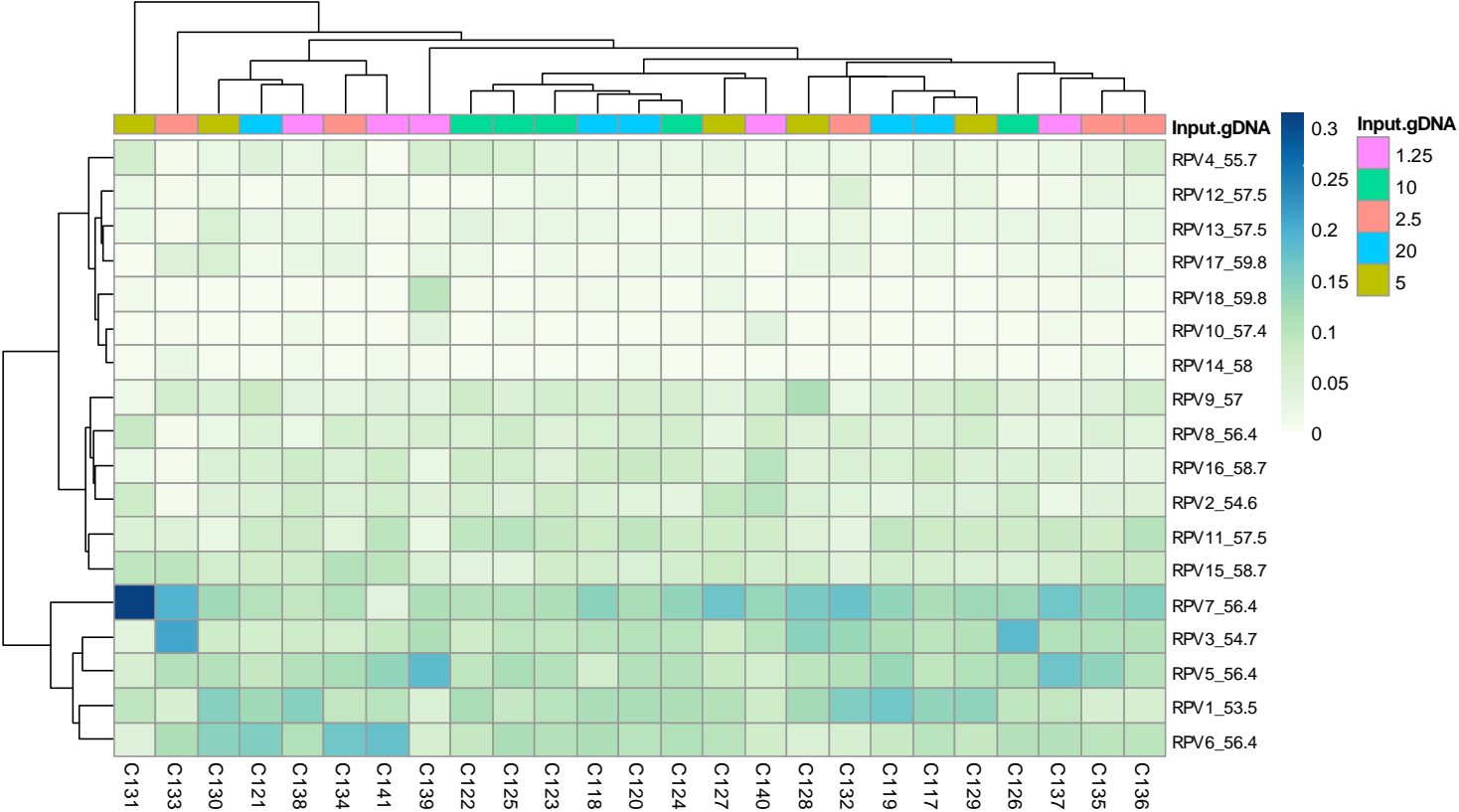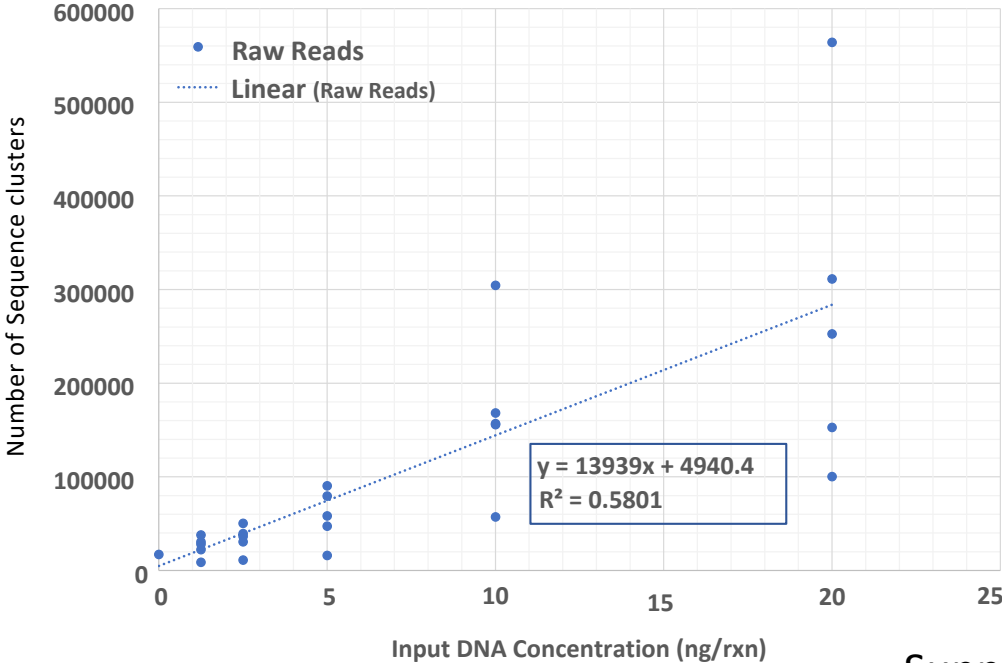

Supplemental Figure S1C

Supplement: Figure S1 — Analyses were performed on rarefied data sets (8,000 sequences per sample), with five technical replicates for each DNA input level (1.25, 2.5, 5, 10 or 20 ng/µl). (A) Genus-level mMDS ordination of microbial community structure using a distance matrix based on Bray–Curtis similarity. No significant differences were observed between all the concentrations (Global ANOSIM: R=-0.03376, p=0.79). Ellipses represent a 95% confidence interval around the centroid. (B) Primer utilization profiles for all primer variants (RPV1 –RPV18), visualized as a heatmap. (C) A positive correlation between input gDNA (1.25, 2.5, 5, 10, 20 ng/µl) and sequence yield was observed. For all input levels, the same gDNA template was used with five technical replicates. All samples were pooled after stage A of DePCR and amplified together using Illumina P5 and P7 primers. Data were rarefied to 8,000 sequences per sample. [file peerj-07-7121-s001.pdf]
